# Correlation Between Low Platelet-to-Lymphocyte Ratio and High Mortality Rates in Adult Trauma Patients With Moderate-to-Severe Brain Injuries

**DOI:** 10.1155/emmi/8099416

**Published:** 2024-12-19

**Authors:** Kang-Wei To, Shiun-Yuan Hsu, Chia-Ying Yu, Yu-Chin Tsai, You-Cheng Lin, Ching-Hua Hsieh

**Affiliations:** ^1^Department of Neurosurgery, Kaohsiung Chang Gung Memorial Hospital and Chang Gung University College of Medicine, Kaohsiung 83301, Taiwan; ^2^Department of Trauma Surgery, Kaohsiung Chang Gung Memorial Hospital and Chang Gung University, Kaohsiung 83301, Taiwan; ^3^Department of Anatomic Pathology, Kaohsiung Chang Gung Memorial Hospital and Chang Gung University College of Medicine, Kaohsiung 83301, Taiwan; ^4^Department of Plastic Surgery, Kaohsiung Chang Gung Memorial Hospital and Chang Gung University, Kaohsiung 83301, Taiwan

## Abstract

**Background:** White blood cell (WBC) subtypes reflect immune and inflammatory conditions in patients. This study aimed to examine the association between the ratio of platelets to WBC subtypes and mortality outcomes in patients with moderate-to-severe traumatic brain injury (TBI).

**Method:** The Trauma Registry System of the hospital was retrospectively reviewed to gather medical records of 2397 adult patients who were hospitalized from 2009 to 2020 and had moderate-to-severe TBI with a head abbreviated injury scale (AIS) score of 3 or higher. The monocyte-to-lymphocyte ratio (MLR), neutrophil-to-lymphocyte ratio (NLR), and platelet-to-lymphocyte ratio (PLR) were compared between the survivors (*n* = 2, 138) and nonsurvivors (*n* = 259). A multivariate logistic regression analysis was performed to investigate the independent effects of the univariate prognostic factors on mortality outcomes. The survival variations among the PLR subgroups were evaluated by the Kaplan–Meier survival analysis including a log-rank test.

**Results:** The PLR of the deceased patients was considerably lower than that of the survivors (129.5 ± 130.1 vs. 153.2 ± 102.1, *p* < 0.001). However, no significant differences were observed in monocyte and neutrophil counts, MLR, or NLR between the deceased and survivor groups. A lower PLR was recognized as an independent risk factor for mortality (odds ratio: 1.26, 95% confidence interval: 1.06–1.51, *p*=0.010). The receiver operating characteristic (ROC) established PLR as the most strong predictor among the three ratios (area under the ROC curve = 0.627, sensitivity = 0.846, and specificity = 0.382, according to the cut-off value = 68.57). When the patient groups were divided by PLR quartile, the Kaplan–Meier analysis showed significantly worse survival in the lowest PLR quartile group (< 83.1) compared with the highest quartile group (≥ 189.1) (*p* < 0.001).

**Conclusion:** Lower PLR is associated with greater mortality in adult patients with moderate-to-severe TBI. PLR may be a valuable measure for classifying mortality risk in this population.

## 1. Introduction

Brain injury is the leading cause of disability and mortality in both developing and developed countries [1]. The early identification of patients with traumatic brain injury (TBI) at high risk of mortality is crucial for optimizing treatment strategies and ensuring effective resource allocation [2]. By recognizing key factors associated with higher mortality, such as severe initial Glasgow Coma Scale (GCS) scores, age, the presence of coagulopathies, and the extent of brain damage revealed through neuroimaging, healthcare providers can prioritize urgent interventions, tailor rehabilitation plans, and make informed decisions about the level of care needed, potentially improving survival rates and long-term outcomes [3–5].

Complete blood count (CBC) is one of the most often conducted tests to evaluate the distribution of blood cell content in trauma patients. Several studies have highlighted the importance of white blood cell (WBC) subtypes and their distribution patterns in determining immune and inflammatory status. For instance, variations in WBC subtype counts, including neutrophils, lymphocytes, and monocytes, can indicate the immune and inflammatory status of individuals with conditions such as prediabetes and type 2 diabetes, revealing the body's response to metabolic disturbances [6]. In patients with trauma, changes in WBC subtypes, particularly in relation to stress-induced hyperglycemia, have been linked to immune responses, highlighting their potential role in influencing patient prognosis and treatment strategies [7, 8]. Furthermore, the computed ratios of platelets and specific WBC subtypes are readily available parameters and can be useful predictors of patient outcomes [9–13]. In these patient populations, several novel predictors of mortality have been documented, such as the neutrophil-to-lymphocyte ratio (NLR) in patients with fracture [14], monocyte-to-lymphocyte ratio (MLR) in osteoporosis patients [15], platelet-to-lymphocyte ratio (PLR) in patients with oncological conditions [16–18], and neutrophil-to-monocyte ratio [19]. These findings reinforce the value of monitoring WBC subtypes as a noninvasive tool for assessing and managing inflammatory and immune responses in patients with trauma [20, 21]. The detection of CBC subtypes is useful in prognosticating the outcomes and predicting the complications of TBI [22–24]. For instance, pediatric patients with elevated NLRs at 24 and 48 h after TBI tend to experience poorer outcomes [25]. NLR can predict the early development of traumatic intracerebral hemorrhage [26]. Additionally, integrating CBC results with radiological findings enhances the predictive accuracy for patient prognosis [22]. Furthermore, an elevated NLR 1 week after a brain contusion and high MLR at admission are important prognostic indicators of unfavorable outcomes over a 6-month period in patients with brain contusions [27].

Severe trauma can lead to substantial blood loss and platelet consumption [28–30], with coagulopathy affecting around one-third of trauma victims [31]. Previous research has shown that 45.5% of patients with serious trauma exhibit platelet hypofunction at admission. Platelet hypofunction was defined as insufficient platelet responsiveness to at least one agonist drug. In addition, during their stay in the intensive care unit, 91.1% of these patients were classified as having platelet hypofunction conditions [32]. Decreased platelet counts are linked to coagulopathy or disseminated intravascular coagulation in trauma patients, both of which heighten the risk of mortality [33–35]. Trauma-induced coagulopathy can cause secondary bleeding, characterized by extensive microvascular hemorrhage that extends beyond the primary injury site [31].

Moreover, a link was established between lymphocyte count and the incidence of multiple organ dysfunction syndrome in patients who have sustained severe trauma [36]. Neutrophils and monocytes can also serve as biomarkers of both immunological status and bone marrow hematopoietic activity [37], which may influence patient outcomes after trauma. Moreover, alterations in the levels of platelet and various subtypes of WBCs would lead to modifications in the MLR, NLR, and PLR in patients with trauma. Given the often accidental and sudden nature of injuries and the unique characteristics of patients with trauma, investigating the associations between platelets; various WBC subtypes; and MLR, NLR, and PLR are linked to the prognosis of critically injured patients with TBI. Due to the low death rate of mild TBI patients, this study focuses on adult trauma patients with moderate-to-severe TBI, defined as a head abbreviated injury scale (AIS) score of ≥ 3. The purpose of this retrospective study was to determine the relationship between MLR, NLR, and PLR and these patients' mortality risk utilizing data from a registered trauma database at a Level I trauma hospital.

## 2. Materials and Methods

### 2.1. Study Population

**Figure 1:**
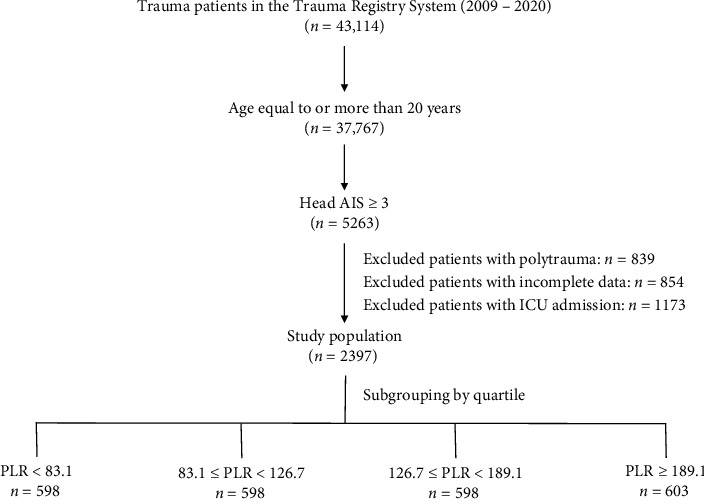
Enrollment and allocation of patients from the registered trauma database in the study.

The Institutional Review Board (IRB) of Chang Gung Memorial Hospital authorized this study (approval number: 201901582B0). The necessity for consent that was informed was waived due to the study's retrospective design. Data on adult trauma patients admitted to the emergency department from January 1, 2009, to December 31, 2020, were extracted from the hospital's Trauma Registry System. Among the 43,114 trauma patients registered during the study period, 37,767 were aged ≥ 20 years. Only patients with a head AIS score of ≥ 3 (moderate-to-severe TBI) were included. After excluding patients with polytrauma (*n* = 839), burns (*n* = 0), ward admission (*n* = 1173), and incomplete data (*n* = 854), only 2397 were evaluated. The patients were categorized into survivors (*n* = 2138) and nonsurvivors (*n* = 259). The patients were further divided into four groups by PLR quartile: PLR < 83.1 (*n* = 598), 83.1 ≤ PLR < 126.7 (*n* = 598), 126.7 ≤ PLR < 189.1 (*n* = 598), and PLR ≥ 189.1 (*n* = 603) (Figure 1). Information on sex, age, comorbidities, GCS score, injury severity score (ISS), and death during the hospitalization was extracted from the hospital's registered trauma database. The analyzed comorbidities encompassed cerebrovascular accident (CVA), hypertension, coronary artery disease (CAD), congestive heart failure, diabetes mellitus, and end-stage renal disease (ESRD). Laboratory analysis of blood samples obtained upon admission to the emergency room determines the count of platelets, neutrophils, monocytes, and lymphocytes of the patients. Subsequently, the MLR, NLR, and PLR were computed based on these results.

### 2.2. Statistical Analysis

The statistical analyses were conducted using IBM SPSS Statistics 23 (IBM Corporation, USA). Participants were categorized based on survival outcomes and PLR quartiles. Categorical variables were assessed using chi-square tests, with odds ratios (ORs) and 95% confidence intervals (CIs) calculated. Continuous variables were presented as mean ± standard deviation, except for GCS and ISS, which were expressed as median and interquartile range (IQR, Q1–Q3). Homogeneity of variance for continuous variables was evaluated using Levene's test, followed by one-way ANOVA with Games–Howell post hoc analysis. Multivariate logistic regression, including variance inflation factor (VIF) calculations, was employed to determine independent mortality risk factors in moderate-to-severe TBI cases. The predictive capabilities of MLR, NLR, and PLR were assessed using the area under the receiver operating characteristic curve (AUC). Youden's index was utilized to establish optimal cutoff values for these ratios [38]. An AUC value close to 1 denotes excellent predictive power, while an AUC value of 0.5 suggests the absence of predictive ability. Additionally, the Kaplan–Meier survival curves were plotted using the SPSS software to compare the cumulative survival rates across the four PLR subgroups over a 200-day period, from the time of blood collection to death or discharge. The Mantel–Cox log-rank test was performed to determine statistical differences in survival distributions across groups. This nonparametric test is commonly used in survival analysis to compare survival curves, accounting for censored data and providing *p* values for pairwise comparisons between the groups. A *p*-value of less than 0.05 was deemed significant.

## 3. Results

### 3.1. Patient and Injury Characteristics

Table 1 shows that sex had no significant effect on fatality rates in this study. Older adults and those with HTN (44.4% vs. 38.0%, *p*=0.045), CAD (14.7% vs. 7.8%, *p* < 0.001), and ESRD (13.1% vs. 2.8%, *p* < 0.001) had higher mortality rates than those in the survival group. Comparative analysis demonstrated that patients in the mortality group presented with significantly compromised neurological status, as evidenced by lower GCS scores (median [IQR]: 5 [3–12] vs. 15 [11–15] in the survival group, *p* < 0.001). Additionally, the mortality group exhibited markedly higher ISS (median [IQR]: 25 [25-25] vs. 16 [16–20] in the survival group, *p* < 0.001). Patients who died exhibited significantly higher lymphocyte counts (2376.4% ± 1886.4% vs. 1855.3% ± 1319.1%, *p* < 0.001) and lower platelet counts (177,891.9 ± 65,157.3/μL vs. 206,899 ± 68,239.5/μL, *p* < 0.001) compared with survivors. Nonetheless, no significant difference was detected in the monocyte and neutrophil counts between the nonsurvivor and survivor cohorts. Deceased patients demonstrated a significantly reduced PLR compared to survivors (129.5 ± 130.1 vs. 153.2 ± 102.1, *p* < 0.001), although no notable variations were found in MLR and NLR between the two cohorts. Additionally, the deceased patients had a significantly shorter hospital stay compared with survivors (9.7 ± 14.2 vs. 14.3 ± 13.9 days, *p* < 0.001)

### 3.2. PLR Subgroup Analysis

Table 2 presents a comparison of the patient characteristics across the four PLR quartile subgroups: PLR < 83.1 (*n* = 598), 83.1 ≤ PLR < 126.7 (*n* = 598), 126.7 ≤ PLR < 189.1 (*n* = 598), and PLR ≥ 189.1 (*n* = 603). The subgroup with the lowest PLR (< 83.1) had a higher proportion of male patients, younger age, lower GCS scores, higher ISSs (with 31.9% having ISS ≥ 25), the longest hospital stay, the highest lymphocyte count, the lowest platelet count, and the highest mortality rate (19.1%). As the PLR quartile increased from < 83.1 to ≥ 189.1, the proportion of men decreased, age increased, the GCS scores increased, the proportion of patients with an ISS of ≥ 25 decreased, the length of hospital stay increased, the lymphocyte count decreased, the platelet count increased, and the mortality rates decreased. The differences in sex, GCS scores, ISSs, length of hospital stay, lymphocyte and platelet counts, and mortality rates across the PLR quartiles were significant (*p* < 0.001). This finding suggests that the lowest PLR quartile was associated with markers of more severe injury and worse outcomes.

### 3.3. Analysis of Risk Factors for Mortality

The univariate logistic regression analysis revealed multiple parameters significantly correlated with death in adult patients with moderate-to-severe TBI (Table 3). These factors included advanced age, pre-existing HTN, CAD, ESRD, lower GCS score, higher ISS, decreased platelet count, elevated lymphocyte count, and decreased PLR. The multivariate logistic regression model revealed a diverse array of independent risk factors significantly associated with mortality. These encompassed patient characteristics (advanced age), pre-existing conditions (CAD & ESRD), injury severity indicators (lower GCS scores, higher ISS), and hematological parameters (increased lymphocyte counts, decreased platelet counts, and lower PLR). Notably, ESRD exhibited the strongest association with mortality risk (OR: 6.39, 95% CI: 3.57–11.44, *p* < 0.001), while CAD presented the second highest risk (OR: 2.05, 95% CI: 1.23–3.41, *p*=0.006). Examination of blood cell parameters yielded significant correlations with mortality outcomes. Higher lymphocyte counts were associated with an increased likelihood of mortality (OR: 1.29, 95% CI: 1.13–1.47, *p* < 0.001), while elevated platelet counts demonstrated a protective effect (OR: 0.42, 95% CI: 0.31–0.57, *p* < 0.001). Additionally, higher PLR were linked to increased mortality risk (OR: 1.26, 95% CI: 1.06–1.51, *p*=0.010).  Table 4 presents the results of the VIF analysis of the multivariate model. All variables showed a VIF value of < 5, with the highest being 2.344 for lymphocytes and the lowest being 1.051 for ESRD. These low VIF values indicate minimal multicollinearity among the independent variables, suggesting the lack of strong correlation between these variables. This strengthens the reliability of the multivariate analysis results and supports the validity of the statistical approach and findings of this study.

### 3.4. Prediction Performance of MLR, NLR, and PLR for Mortality

**Figure 2:**
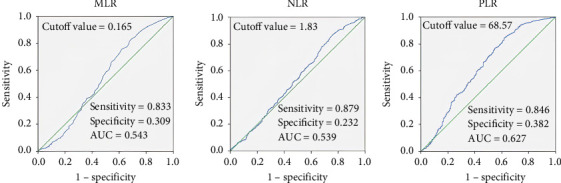
Performance of MLR, NLR, and PLR in predicting mortality determined by the area under the receiver operating characteristic curve.

To compare the predictive efficacy of MLR, NLR, and PLR, receiver operating characteristic (ROC) curves were constructed and analyzed (Figure 2). The analysis revealed that PLR possessed the highest discriminative capacity, with an AUC of 0.627. At the threshold value of 68.57, PLR achieved a sensitivity of 0.846 and a specificity of 0.382. The NLR showed the highest sensitivity (0.879) but the lowest specificity (0.232), with an AUC of 0.539 and a cutoff value of 1.83. MLR showed a similar performance to NLR, with an AUC of 0.543, a sensitivity of 0.833, a specificity of 0.309, and a cutoff value of 0.165. These results suggest that PLR was the most effective predictor of outcomes assessed in this study.

### 3.5. Analysis of the Kaplan–Meier Survival Curves

**Figure 3:**
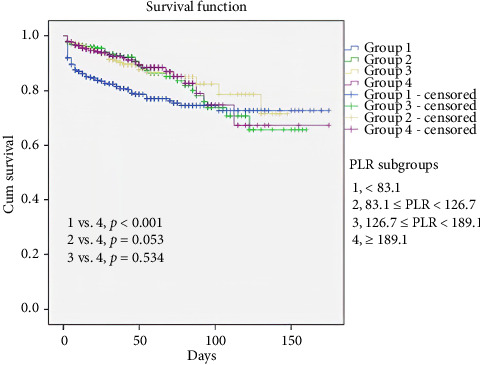
Analysis of the Kaplan–Meier survival curves by patient subgroups according to PLR quartile.

Survival curves demonstrated clear stratification based on PLR subgroups (Figure 3). The lowest PLR group (< 83.1) exhibited significantly worse survival compared with the highest PLR group (≥ 189.1) (*p* < 0.001). The second lowest PLR group (83.1 ≤ PLR < 126.7) demonstrated lower survival compared with the highest group, although marginally significant (*p*=0.053). In the meantime, no significant difference was detected between the two groups exhibiting the highest PLR (*p*=0.534). These results suggest that lower PLR values, particularly those below 83.1, are associated with poorer survival outcomes in this patient population, with survival rates improving as the PLR increases.

## 4. Discussion

Our findings demonstrate a notable correlation between PLR and mortality outcomes in adults presenting with moderate-to-severe TBI. Furthermore, multivariate logistic regression modeling confirmed that a reduced PLR independently contributes to increased mortality risk in this cohort. The ROC curves revealed that the PLR had the strongest overall performance in predicting mortality outcomes compared with the MLR and NLR. When the patients were divided by PLR quartile, the Kaplan–Meier analysis showed significantly worse survival in the lowest PLR group (< 83.1) compared with the highest PLR group (≥ 189.1) (*p* < 0.001). Furthermore, deceased patients exhibited a lower platelet count and an elevated lymphocyte count compared with survivors. The study examined the PLR, a hematological index derived from the relative counts of platelets and lymphocytes. Notably, lower PLR values, indicative of reduced platelet counts in conjunction with elevated lymphocyte counts, were found to be significantly associated with higher mortality rates in the patient population.

Platelets are not the only blood components that are affected by severe trauma. In conjunction with decreased platelet count, WBCs get moving during central hypovolemia, resulting in a relative elevation of WBC count, referred to as leukocytosis [39]. In these patients, a higher lymphocyte count can indicate hypovolemia [36], which may result in hypotension, decreased cerebral blood flow, and impaired cerebral perfusion. Individuals who exhibited reduced cerebral blood flow within the initial 7 days following head injury showed elevated mortality rates and poorer recovery [40]. Leukocytosis involves lymphocytes, monocytes, and neutrophils. In this study, neither monocytes and neutrophils nor the MLR or NLR showed no significant differences between nonsurvivors and survivors. Increased lymphocyte count contributes to the downregulation of inflammatory responses. However, neutrophils may be associated with the amplification of inflammatory reactions [41–43]. The acute increase in lymphocyte count in the early phase of injury is attributed to the response of patients to trauma and is related to injury severity and mortality [44, 45].

A lower PLR has been recognized as an indicator of adverse outcomes in patients with intracerebral hemorrhage, as evidenced in the literature [46–48]. Idowu, Oyeleke, and Vitowanu found that a low PLR upon admission was not only indicative of coagulopathy, platelet dysfunction, and thrombocytopenia but also related to unfavorable outcomes in patients with chronic subdural hematoma [46]. This reduction in PLR can be attributed to the substantial release of tissue factors following severe TBI, which triggers and enhances the coagulation cascade, leading to platelet depletion and a consequent reduction in PLR [47]. Additionally, a low PLR serves as a circulatory indicator related to cerebral hemorrhage following endovascular thrombectomy [48].

Some studies have shown contradictory findings concerning the relevance of PLR in adult patients with head trauma. Zhang and Shen [49] performed a retrospective analysis of 183 individuals diagnosed with cerebral hemorrhage. A high PLR at admission was found to correlate with lower GCS scores, signifying a poorer prognosis. Additionally, as a more precise indicator of elevated inflammation levels, PLR outperformed platelet and lymphocyte counts in predicting neurological outcomes in individuals with cerebral hemorrhage. Recent research by Yun et al. [50] has elucidated the potential of PLR as an inflammatory biomarker for prognosticating clinical outcomes following aneurysmal subarachnoid hemorrhage. Their findings indicated that PLR values exceeding 130 were significantly associated with poor clinical outcomes 90 days post-incident. While platelets may not be directly implicated in coagulopathy, they play a crucial role in inflammatory processes and immunomodulation. This influence is exerted through enhanced cytokine production and complex interactions with other cellular components of the immune system [51, 52]. A dysregulated PLR may reflect an imbalance in thrombocyte activity and an exacerbated inflammatory response.

This study's findings should be interpreted in light of certain methodological limitations. The retrospective design inherently carries a risk of selection bias, which may have been exacerbated by the exclusion of cases with incomplete data. Furthermore, the study population did not capture individuals who died immediately upon emergency room arrival, potentially affecting the comprehensiveness of the mortality analysis. Additionally, the evaluation focused solely on death rates in the hospital setting without considering long-term mortality. Furthermore, the levels of platelets and different WBC subtypes can vary significantly throughout the therapeutic process, potentially affecting the resuscitation outcomes. Although blood samples were collected from patients upon admission to the emergency room, the likelihood of blood transfusion was reduced. However, the fluid challenge administered during resuscitation may not have adhered to a specific protocol, and the exact volume of fluid administered has not been documented. This lack of adherence to a defined protocol and uncertainty regarding the volume of fluids administered could introduce bias when measuring study outcomes. Fourth, the study did not categorize TBI into specific subtypes, such as traumatic intracerebral hemorrhage or epidural and subdural hematoma, which may have different impacts on the assessment. Finally, the generalizability of the study findings is potentially compromised by the single-center nature of the investigation, which was conducted exclusively at a metropolitan trauma center. This limitation underscores the need for caution when attempting to apply these results to institutions with different patient populations, resource allocations, or treatment paradigms.

## 5. Conclusions

This study found that lower PLRs were associated with higher fatality rates in adult patients with moderate-to-severe TBI. PLR is a simple and uncomplicated tool that can be used to stratify the risk of death in these patients.

## Figures and Tables

**Table 1:** General and injury characteristics of survivors and nonsurvivors.

Variables	Death *n* = 259	Survival *n* = 2138	OR (95% CI)	*p*
Sex				0.057
Male, *n* (%)	172 (66.4)	1289 (60.3)	1.30 (0.99–1.71)	
Female, *n* (%)	87 (33.6)	849 (39.7)	0.77 (0.59–1.01)	
Age, years	64.3 ± 17.9	59.4 ± 18.8	—	< 0.001
Comorbidities				
CVA, *n* (%)	20 (7.7)	153 (7.2)	1.09 (0.67–1.76)	0.740
HTN, *n* (%)	115 (44.4)	812 (38.0)	1.30 (1.01–1.69)	0.045
CAD, *n* (%)	38 (14.7)	166 (7.8)	2.04 (1.40–2.98)	< 0.001
CHF, *n* (%)	2 (0.8)	16 (0.7)	1.03 (0.24–4.51)	0.967
DM, *n* (%)	57 (22.0)	448 (21.0)	1.06 (0.78–1.45)	0.695
ESRD, *n* (%)	34 (13.1)	59 (2.8)	5.33 (3.42–8.30)	< 0.001
GCS, median (IQR)	5 (3–12)	15 (11–15)	—	< 0.001
3–8	179 (69.1)	395 (18.5)	9.87 (7.42–13.13)	< 0.001
9–12	20 (7.7)	262 (12.3)	0.60 (0.37–0.96)	0.033
13–15	60 (23.2)	1481 (69.3)	0.13 (0.10–0.18)	< 0.001
ISS, median (IQR)	25 (25–25)	16 (16–20)	—	< 0.001
< 16	4 (1.5)	368 (17.2)	0.08 (0.03–0.20)	< 0.001
16–24	54 (20.8)	1494 (69.9)	0.11 (0.08–0.16)	< 0.001
≥ 25	201 (77.6)	276 (12.9)	23.38 (17.01–32.14)	< 0.001
Monocytes (10^6^/L)	630.3 ± 416.3	590.7 ± 348.6	—	0.143
Neutrophils (10^6^/L)	9588.6 ± 5310.7	9679.2 ± 4973.7	—	0.783
Lymphocytes (10^6^/L)	2376.4 ± 1886.4	1855.3 ± 1319.1	—	< 0.001
Platelets (10^6^/L)	177891.9 ± 65157.3	206,899.0 ± 68239.5	—	< 0.001
MLR	0.4 ± 0.4	0.4 ± 0.3	—	0.750
NLR	7.2 ± 6.4	7.7 ± 6.5	—	0.235
PLR	129.5 ± 130.1	153.2 ± 102.1	—	0.001
Hospital stay (days)	9.7 ± 14.2	14.3 ± 13.9	—	< 0.001

Abbreviations: CAD, coronary artery disease; CHF, congestive heart failure; CI, confidence interval; CVA, cerebrovascular accident; DM, diabetes mellitus; ESRD, end-stage renal disease; GCS, Glasgow Coma Scale; HTN, hypertension; IQR, interquartile range; ISS, injury severity score; MLR, monocyte-to-lymphocyte ratio; NLR, neutrophil-to-lymphocyte ratio; OR, odds ratio; PLR, platelet-to-lymphocyte ratio.

**Table 2:** Comparison among various PLR subgroups divided by quartile.

Variables	PLR subgroup				
	< 83.1 *n* = 598	83.1 ≤ PLR < 126.7 *n* = 598	126.7 ≤ PLR < 189.1 *n* = 598	≥ 189.1 *n* = 603	*p*
Sex					0.001
Male, *n* (%)	401 (67.1)∗	364 (60.9)	359 (60.0)	337 (55.9)	
Female, *n* (%)	197 (32.9)∗	234 (39.1)	239 (40.0)	266 (44.1)	
Age, years	56.2 ± 18.7∗	60.2 ± 19.0	61.3 ± 18.5	61.9 ± 18.4	< 0.001
Comorbidities					
CVA, *n* (%)	30 (5.0)	45 (7.5)	49 (8.2)	49 (8.1)	0.111
HTN, *n* (%)	193 (32.3)	246 (41.1)	258 (43.1)	230 (38.1)	0.001
CAD, *n* (%)	37 (6.2)	57 (9.5)	60 (10.0)	50 (8.3)	0.080
CHF, *n* (%)	5 (0.8)	6 (1.0)	3 (0.5)	4 (0.7)	0.770
DM, *n* (%)	105 (17.6)	142 (23.7)	143 (23.9)	115 (19.1)	0.010
ESRD, *n* (%)	16 (2.7)	34 (5.7)	21 (3.5)	22 (3.6)	0.049
GCS, median (IQR)	11 (6–15)∗	15 (10–15)	15 (12–15)	15 (11–15)	< 0.001
3–8	244 (40.8)∗	124 (20.7)	103 (17.2)	103 (17.1)	< 0.001
9–12	88 (14.7)	67 (11.2)	59 (9.9)	68 (11.3)	0.060
13–15	266 (44.5)∗	407 (68.1)	436 (72.9)	432 (71.6)	< 0.001
ISS, median (IQR)	20 (16–25)∗	16 (16–20)	16 (16–20)	16 (16–20)	< 0.001
< 16	60 (10.0)∗	97 (16.2)	108 (18.1)	107 (17.7)	< 0.001
16–24	347 (58.0)∗	397 (66.4)	398 (66.6)	406 (67.3)	0.002
≥ 25	191 (31.9)∗	104 (17.4)	92 (15.4)	90 (14.9)	< 0.001
Hospital stay (days)	15.8 ± 14.8∗	13.7 ± 13.4	13.2 ± 14.8	12.5 ± 12.9	< 0.001
Lymphocyte (10^6^/L)	3591.8 ± 1702.9∗	1864.8 ± 671.0∗	1316.4 ± 391.0∗	882.1 ± 335.1	< 0.001
Platelet (10^6^/L)	189933.1 ± 74462.3∗	193458.2 ± 64823.7∗	202715.7 ± 57543.0∗	228743.0 ± 69293.1	< 0.001
Mortality, *n* (%)	114 (19.1)∗	50 (8.4)	48 (8.0)	47 (7.8)	< 0.001

Abbreviations: AOR, adjusted odds ratio; CAD, coronary artery disease; CHF, congestive heart failure; CI, confidence interval; CVA, cerebral vascular accident; DM, diabetes mellitus; ESRD, end-stage renal disease; GCS, Glasgow Coma Scale; HTN, hypertension; IQR, interquartile range; ISS, injury severity score; OR odds ratio; PLR, platelet-to-lymphocyte ratio.

^∗^Indicated a significant difference when compared with the group of PLR ≥ 189.1.

**Table 3:** Analysis of the independent risk factors for the mortality outcome using univariate and multivariate logistic regression.

Variables	Univariate analysis		Multivariable analysis	
	OR (95% CI)	*p*	OR (95% CI)	*p*
Male	1.30 (0.99–1.71)	0.057	—	—
Age	1.02 (1.01–1.02)	< 0.001	1.03 (1.02–1.04)	< 0.001
HTN	1.30 (1.01–1.69)	0.045	1.14 (0.80–1.64)	0.464
CAD	2.04 (1.40–2.98)	< 0.001	2.05 (1.23–3.41)	0.006
ESRD	5.33 (3.42–8.30)	< 0.001	6.39 (3.57–11.44)	< 0.001
GCS	0.76 (0.73–0.78)	< 0.001	0.80 (0.77–0.84)	< 0.001
ISS	1.29 (1.25–1.33)	< 0.001	1.21 (1.17–1.26)	< 0.001
Lymphocytes	1.24 (1.15–1.34)	< 0.001	1.29 (1.13–1.47)	< 0.001
Platelets	0.51 (0.41–0.62)	< 0.001	0.42 (0.31–0.57)	< 0.001
PLR	0.76 (0.64–0.89)	0.001	1.26 (1.06–1.51)	0.010

Abbreviations: CAD, coronary artery disease; CI, confidence interval; ESRD, end-stage renal disease; GCS, Glasgow Coma Scale; HTN, hypertension; ISS, injury severity score; OR, odds ratio; PLR, platelet-to-lymphocyte ratio.

**Table 4:** Variance inflation factor between the variables.

	Standardized coefficients			Collinearity statistics	
	Beta	*t*	*p*	Tolerance	VIF
(Constant)		14.568	< 0.001		
Male	0.015	0.815	0.415	0.943	1.060
Age	−0.080	−3.567	< 0.001	0.670	1.493
HTN	0.032	1.516	0.130	0.769	1.300
CAD	0.042	2.191	0.029	0.911	1.097
ESRD	0.139	7.377	< 0.001	0.951	1.051
GCS	0.319	14.948	< 0.001	0.740	1.352
ISS	−0.246	−11.956	< 0.001	0.790	1.265
Lymphocytes	−0.089	−3.183	0.001	0.427	2.344
Platelets	0.120	5.158	< 0.001	0.617	1.621
PLR	−0.063	−2.422	0.016	0.492	2.031

Abbreviations: CAD, coronary artery disease; CI, confidence interval; ESRD, end-stage renal disease; GCS, Glasgow Coma Scale; HTN, hypertension; ISS, injury severity score; PLR, platelet-to-lymphocyte ratio; VIF, variance inflation factor.

## Data Availability

The data that support the findings of this study are available from the Trauma Registry System of Kaohsiung Chang Gung Memorial Hospital but restrictions apply to the availability of these data, which were used under institutional approval for the current study, and so are not publicly available. Data are however available from the corresponding author, Prof. Ching-Hua Hsieh (m93chinghua@gmail.com), upon reasonable request and with the following conditions: 1. A formal research proposal must be submitted. 2. Approval from the Institutional Review Board of Institute must be obtained. 3. A data sharing agreement must be signed. 4. Data will be provided in de-identified format only. 5. Use must comply with original IRB approval (number: 201901582B0). 6. Any secondary analysis must acknowledge the original data source. Restrictions on data sharing are in place to protect patient privacy and comply with institutional policies.
